# Self-assembled membrane manufactured by metal–organic framework (UiO-66) coated γ-Al_2_O_3_ for cleaning oily seawater†

**DOI:** 10.1039/c9ra00521h

**Published:** 2019-04-05

**Authors:** Cunlong Li, Yuqing Zhang, Ming Yong, Wei Liu, Jiaqi Wang

**Affiliations:** School of Chemical Engineering and Technology, Tianjin University Tianjin 300072 PR China zhangyuqing@tju.edu.cn +86-22-27403389 +86-13-602077041

## Abstract

In order to effectively clean oily seawater with anionic polyacrylamide (APAM), UiO-66 coated γ-Al_2_O_3_ (UA) composites were firstly synthesized using γ-Al_2_O_3_ as a template to induce the growth of high hydrophilic UiO-66 on its surface to form a uniform UA self-assembled membrane. The UA composites and self-assembled membrane were characterized and analyzed. Also, the membrane performance was investigated. The results show that the hydrophilicity of particles is enhanced with the water contact angle decreasing from 39.8° (γ-Al_2_O_3_ particles) to 26.2° (UA composites) by introducing the UiO-66 coating. Moreover, the UA self-assembled membrane performs attractive water yield and separation performance. The oil concentration in the permeate treated by the first class of UA self-assembled membrane declines apparently from 91.22 to 18.90 mg L^−1^, while the water yield is as high as 657.89 L m^−2^ h^−1^. The reclaimed separation experiments show that the membrane materials could be recycled by calcination at 200 °C and hydraulic cleaning, which gives the material potential application in cleaning oily seawater.

## Abstract 2

The self-assembled membrane manufactured by UA composites exhibits excellent separation performance and water yield in the treatment of oily seawater.

## Graphical abstract

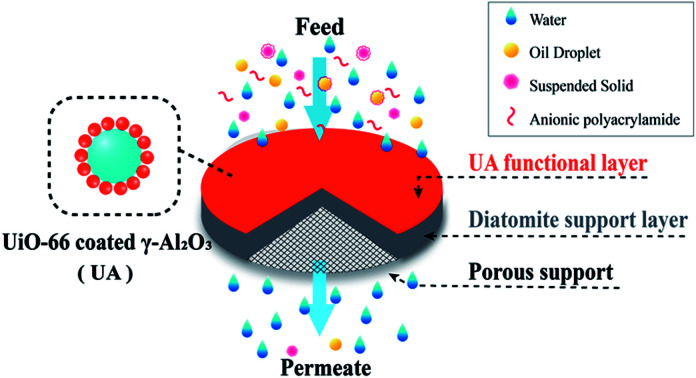

## 1. Introduction

Ultrafiltration (UF) membranes suffer from inherent hydrophobicity, membrane fouling and limited flux.^1,2^ In particular, when treating oily seawater from oil fields, linear APAM molecules along with their carried oil droplets readily pass through a membrane channel.^3^ Accordingly, water quality of a membrane permeate would deteriorate. Even worse, UF membranes would be severely fouled and damaged. To date, dynamic membranes (DM) are an emerging membrane that dynamically come into being *in situ* by filtering solutions containing either inorganic or organic materials through a porous substrate.^4,5^ In contrast to conventional membranes, DM distinguishes itself by having high flux, satisfactory retention efficiency and convenient operation.^6^ Once the membrane is severely fouled, the membrane layer can be removed, and then a new membrane layer can be formed again. However, application of DM is restricted due to the lack of functional groups, abundant material category, uniform spherical shape, appropriate particle size, uniform membrane and the state-of-art membrane technologies.^7^

**Fig. 1:**
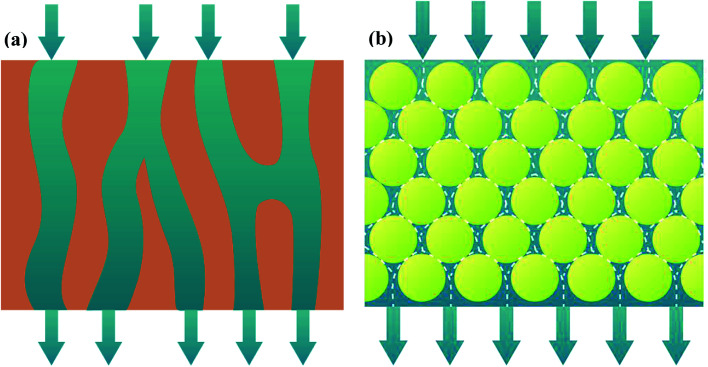
Schematic diagram of membrane channels and fluid flow: polymeric membrane (a); self-assembled membrane (b).

Layer-by-layer (LbL) technology is well-known for its tunable properties. To be specific, it has the ability to regulate coated layer structure and ease of creating composite materials.^8^ Hence, LbL technology supplies the attractive strategies of self-assembled membranes. The self-assembled membranes comprise porous support, support layer and functional layer. The interactions between functional groups of support layer and functional materials through electric charges or hydrogen bonding play an important role in forming uniform self-assembled membrane.^9–11^ Especially, channels of self-assembled membrane are curved. As fluid flows into these curved channels, the interfacial area and turbulence of fluid are apparently enhanced, promoting mass transfer coefficient of liquid phase (shown in Fig. 1).

Although the self-assembled membrane formed by a type of material exhibits passable performance, researches indicated that the self-assembled membrane formed by several types of materials including functional layers shows improved performance.^12,13^ Based on previous research, γ-Al_2_O_3_ is highly effective to eliminate APAM in waste-water through strong electric adsorption.^14^ Therefore, Zhang *et al.*^15^ successfully prepared phosphorylated Zr_*x*_Si_1−*x*_O_2_/Al_2_O_3_ (PZSA) composite, then the PZSA particles served as functional layer, and PZSA self-assembled membrane was formed. Unlike Zr_*x*_Si_1−*x*_O_2_ and phosphorylated Zr_*x*_Si_1−*x*_O_2_ (SZP) self-assembled membrane, PZSA self-assembled membrane performs better separation and water cleaning capability. Nevertheless, the preparation process of PZSA is complicated, tedious and time-consuming. Resulting from the limited Lewis acid sites, hydroxyls and adsorption capability of PZSA particles, PZSA self-assembled membrane exhibits limited cleaning efficiency and water yield for oily seawater. It is therefore indispensable to design new functional materials to form self-assembled membrane with attractive cleaning efficiency and water yield.

Recently, metal–organic frameworks (MOFs) have demonstrated significant research interest in functional material domains. MOFs are connected by linking metal ions or clusters with organic linkers through coordination bonds.^16^ Most MOFs possess high surface area, well-defined pores, selective adsorption properties and open metal sites in the skeleton.^17^ In comparison to other porous adsorbents, MOFs have stronger adsorption and removal abilities for various contaminants. This phenomenon mainly attributes to the central metals and coordinatively unsaturated sites.^18^ These collectively improve the interaction between the MOFs and adsorbate. Haque *et al.*^19^ reported that MOF-235 was effective for the removal of harmful dyes (anionic dye methyl orange (MO) and cationic dye methylene blue (MB)) derived from sewage. The adsorption capacities of MOF-235 for MO and MB by MOF-235 were 477 and 187 mg g^−1^, which are 43 and 7 times than that of activated carbon, respectively. But then, the shape and size distribution of MOFs are important to form uniform self-assembled membranes with well-ordered channels and adjustable pore size. Therefore, uniform spherical MOFs composites are proposed *via* combining MOFs with spherical substrate. MOFs composites combine the advantage of γ-Al_2_O_3_ and UiO-66, providing synergistic effect. For example, Fu *et al.*^20^ fabricated monodisperse ZIF-8@SiO_2_ core–shell microspheres as the stationary phase for HPLC application. As the synergistic effects of good column packing properties of silica spheres and separation ability of ZIF-8 nanocrystals, the ZIF-8@SiO_2_ core–shell microspheres packed column have fast and high-resolution separation of endocrine-disrupting chemicals and pesticides under low column backpressure. Zhao *et al.*^21^ synthesized magnetic Fe_3_O_4_/Cu_3_(BTC)_2_ composites as sorbent merges advantages of Cu_3_(BTC)_2_ and Fe_3_O_4_ nanoparticles. It possesses large adsorption capacity, rapid removal and easy separation for the solid phase, making it become an attractive sorbent in waste-water treatment.

Based on above mentioned studies and analyses, if UA microsphere is prepared using an *in situ* growth method, and then employed them as functional layer to form UA self-assembled membrane on porous support for cleaning oily seawater, which could show the attractive cleaning efficiency and water yield. This is because γ-Al_2_O_3_ is highly effective to adsorb APAM and UiO-66 possesses high hydrophilicity, high specific surface area, excellent moisture stability and controllable structures.^22–24^ In addition, UiO-66 can adsorb some contaminants, such as *n*-alkanes, iso-alkanes, aromatic hydrocarbons in the oily seawater by van der Waals, hydrogen bonding and π–π interactions.^25–27^ γ-Al_2_O_3_ has been reported to form strong interaction with carboxylic acid.^28,29^ It is more likely for carboxylated-based MOFs to be coated onto γ-Al_2_O_3_ surface. So, UA composites exhibit attractive adsorption capacity, high hydrophilicity and the adsorption mechanism is shown in Fig. 3. Thereby, the UA self-assembled membrane with hydrophilicity, selective adsorption and high water yield is desired for oily seawater treatment.

In this paper, to effectively clean oily seawater with APAM, positively charged γ-Al_2_O_3_ microspheres were synthesized and then porous high hydrophilic UiO-66 was firstly coated on the surface of γ-Al_2_O_3_ microspheres to prepare UA composites. Finally, UA self-assembled membrane was formed on porous support. UA composites were characterized and analyzed, and the membranes formed under the optimal conditions were used for cleaning oily seawater. Consequently, the UA self-assembled membrane will exhibit promising alternatives in the oily seawater treatment.

## 2. Experimental

### 2.1 Materials and reagents

Aluminum sulphate (Al_2_(SO_4_)_3_·18H_2_O, AR grade, 99.0%) and anhydrous methanol (CH_3_OH, AR grade, 99.5%) were bought from Real & Lead chemical Co., Ltd. Urea (H_2_NCONH_2_, GR grade, 99.0%) and anhydrous ethanol were obtained from Tianjin Guangfu Fine Chemical Co., Ltd. Terephthalic acid (H_2_BDC, AR grade, 99%) was provided by Heowns Biochem Technologies Co., Ltd. Zirconium chloride (ZrCl_4_, AR grade, 98%) and *N*,*N*′-dimethylformamide (DMF, AR grade, 99.5%) were supplied by Aladdin. Diatomite was obtained from Qingdao Sanxing Diatomite Co., Ltd. The detailed composition of diatomite is shown in Table S1.† All reagents and chemicals were used as received.

### 2.2 Synthesis of UA composites

γ-Al_2_O_3_ was prepared according to a previous report.^30^ Briefly, a 70 mL mixed solution was prepared by dissolving Al_2_(SO_4_)_3_·18H_2_O and urea in deionized water. The concentration of Al^3+^ and urea were 0.1 mol L^−1^ and 1.0 mol L^−1^, respectively. Then the mixture was transferred into a Teflon-lined autoclave (100 mL capacity), which was sealed and heated at certain temperature for certain time. After being cooled to room temperature, the precursors of alumina were collected by centrifugation and washed 3 times. Finally, the precursors were dried in vacuum oven at 60 °C overnight, and the solids were calcined at 500 °C for 4 h to transform them into γ-Al_2_O_3_.

**Fig. 2:**
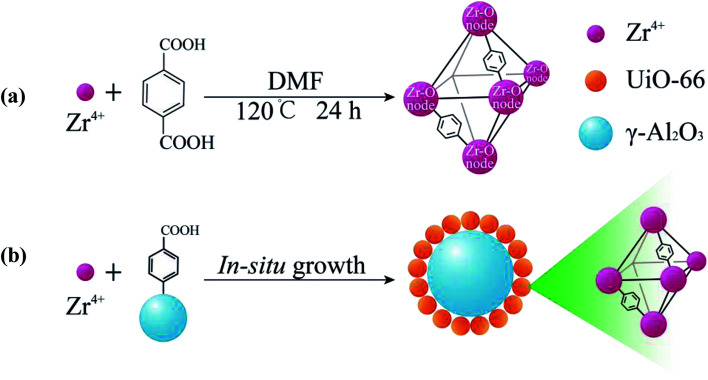
Illustration of the synthesis process of UiO-66 (a) and the *in situ* growth of UA composites (b).

UA composites were prepared by *in situ* growth of UiO-66 onto the surface of γ-Al_2_O_3_ microspheres and the synthesis procedure is shown in Fig. 2(b). To fabricate the UA composites, a given amount of γ-Al_2_O_3_ was dispersed in 250 mmol DMF that had dissolved 0.5 mmol H_2_BDC. After stirring for 10 min, 0.5 mmol ZrCl_4_ and 0.5 mmol deionized water were added to the mixture. The mixture was stirred for 10 min and transferred into a Teflon-lined autoclave and heated at 120 °C for 24 h in an oven. When cooled down, the as-prepared mixture was repeatedly centrifuged and washed several times with DMF and anhydrous methanol, and then dried at 120 °C overnight for further use. For comparison, pure UiO-66 nanoparticles were prepared with the same process without adding γ-Al_2_O_3_ microspheres (Fig. 2(a)) and the details of synthesis route are shown in ESI.†

### 2.3 Formation of UA self-assembled membrane

**Fig. 3:**
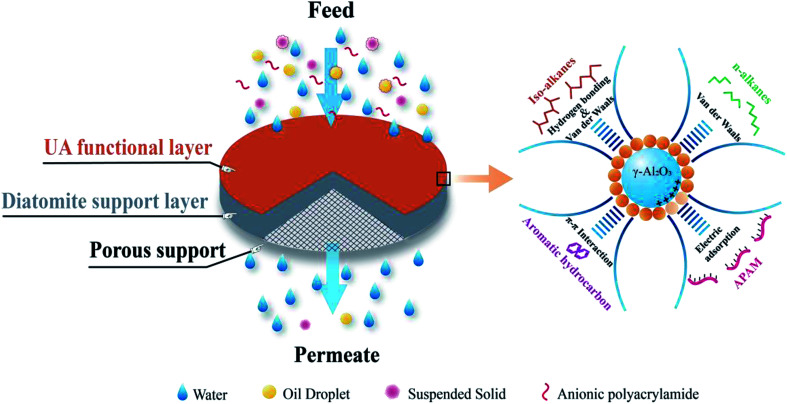
The plausible mechanism of UA self-assembled membrane for treating oily seawater.

The self-assembled membrane was formed using a self-assembled membrane device (Fig. S1, ESI†) made in our laboratory. Before the experiment, the device was thoroughly cleaned to ensure that the pure water flux of the porous support was restored completely. In order to form a more uniform membrane, a liquid distributor and a stainless steel mesh (80 mesh) were used, and the stainless steel mesh was placed between the liquid distributor and membrane to act as a liquid redistributor. The forming process of self-assembled membrane is consistent with our previous studies.^15^ UA self-assembled membrane was formed in two steps. The first step was to pump a diatomite solution (3.0 g L^−1^, 500 mL) to form diatomite support layer on porous support, while the next step was to circulate an UA solution to form UA functional layer on the diatomite support layer. Finally, UA self-assembled membrane was formed. The schematic diagram of UA self-assembled membrane and its plausible mechanism for treating oily seawater are shown in Fig. 3.

### 2.4 Treatment of UA self-assembled membrane for oily seawater

**Fig. 4:**
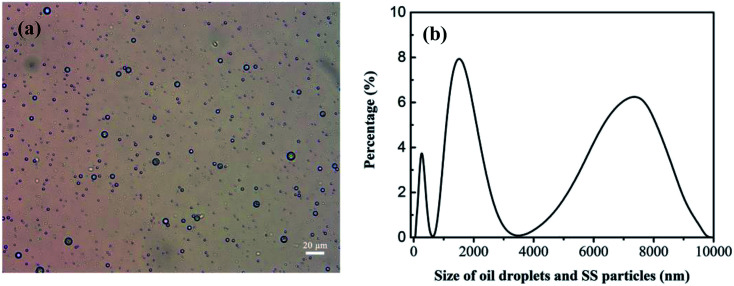
Size distribution of oil droplets and SS particles in oily seawater as feed solution. Optical microscope image (a). The nano particle size analyzer (b).

The oily seawater was obtained from Liaohe Oilfield of China National Petroleum Corporation and pretreated by gravity settling, flocculation precipitation, gas flotation and inclined plate precipitation. Characteristic parameters of raw oily seawater after pretreatment are shown in Table 1. The size and morphology of oil droplets and suspended solid were measured by metallurgical microscope (10XB-PC, 001441) and the nano particle size analyzer (Nano ZS, 030220). Because oil droplets and SS particles coexist in the feed and permeate, the size distribution of oil droplets and SS particles is tested together. As shown in Fig. 4, the size of oil droplet and suspended solid in feed solution is mainly distributed at 1000–2800 nm and 4500–9000 nm.

**Table 1:** Characteristic parameters of raw oily seawater

Seawater quality parameters (units)	Concentration	The discharge and recycle standard in China (SY/T5329-94)
Oil concentration (mg L^−1^)	91.22	8.00
Suspended solid (mg L^−1^)	53.75	3.00
Turbidity (NTU)	15.53	—
Conductivity (ms cm^−1^)	8.71	—
TOC (mg L^−1^)	324.80	—
Temperature (°C)	25.00	—
pH	6.80	—

After formation of the self-assembled membrane, the oily seawater was treated and repeatedly cycled in the self-assembled membrane system for a certain time to obtain permeate water. The permeate water quality should satisfy the discharge and recycle standard in China (SY/T5329-94, concentration of oil ≤ 8 mg L^−1^, suspended solid (SS) ≤ 3 mg L^−1^). As shown in Fig. 3, UA self-assembled membrane was used for treating oily seawater. In order to obtain optimal treating effects, some parameters such as membrane-forming pressure, the molar ratio of γ-Al_2_O_3_ to ZrCl_4_, membrane running time and treatment classes on permeate water quality were studied.

### 2.5 Characterization of UA composites and UA self-assembled membrane

#### 2.5.1 SEM studies

The morphology of γ-Al_2_O_3_ particles, UA composites and UA self-assembled membrane were observed and analyzed using a Hitachi S-4800 scanning electron microscope (SEM) with a resolution of 1 nm.

#### 2.5.2 FTIR studies

FTIR spectra of γ-Al_2_O_3_ particles, UiO-66 particles and UA composites were recorded on a Nexus 470 spectrometer (Thermo Nicolet Corporation, USA) in the frequency range of 4000–400 cm^−1^ with a resolution of 0.1 cm^−1^.

#### 2.5.3 XRD studies

Crystal type of γ-Al_2_O_3_ particles, UiO-66 particles and UA composites were conducted by a D/MAX 2500 X-ray diffraction (XRD, Rigaku Corporation, Japan) with characteristic rays of Co Kα (*λ* = 0.17890 nm). Data were collected in the 2*θ* range of 5–80°.

#### 2.5.4 Water contact angle measurement

Hydrophilic property of the functional materials were evaluated by water contact angles. The water contact angles were measured by a CAS-OCA20 dynamic contact angle equipment (Dataphysics Corporation, Germany).

### 2.6 Measurement methods

#### 2.6.1 Measurement of instantaneous flux

Instantaneous flux is calculated by eqn (1):1
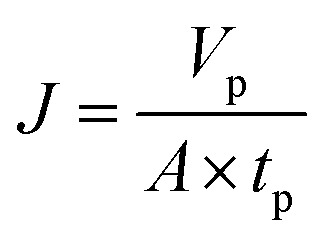
where *J* is the instantaneous flux (L m^−2^ h^−1^), *V*_p_ is the volume of permeate water (L), *t*_p_ is the operating time (h) for obtaining *V*_p_ permeate water, *A* is the effective membrane area (0.00152 m^2^).

#### 2.6.2 Measurement of water yield

Water yield is calculated by eqn (2):2
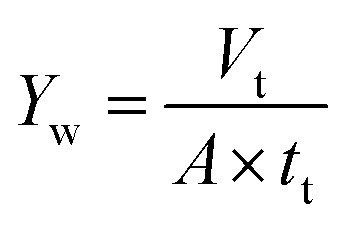
where *Y*_w_ is the water yield (L m^−2^ h^−1^), *V*_t_ is the total volume of oily seawater treated by self-assembled membrane (L), *t*_t_ is membrane running time (h), *A* is the effective membrane area (0.00152 m^2^).

#### 2.6.3 Self-assembled membrane resistance study

The membrane resistance is measured during cleaning process by the application of Darcy's law:^31^3
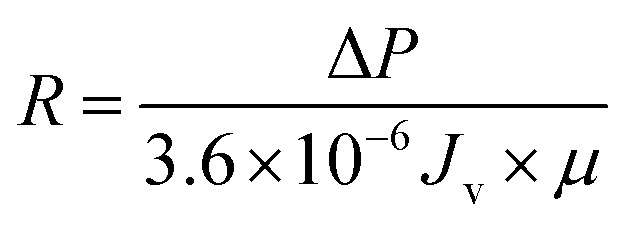
4
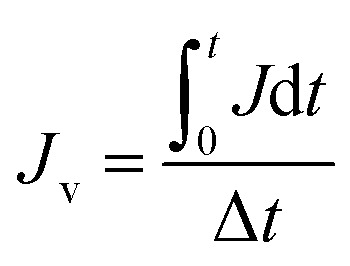
where *R* is the membrane resistance (m^−1^); Δ*P* is operating pressure (Pa); *J*_v_ is the average flux (L m^−2^ h^−1^); and *μ* is the viscosity of oily seawater (Pa s).

#### 2.6.4 Measurement of flux recovery ratio

Flux recovery ratio was calculated by eqn (5):5
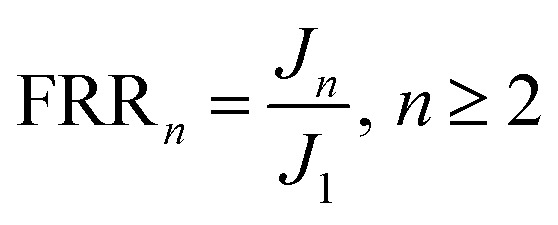
where FRR_*n*_ is the flux recovery ratio (%) as *n* cycles treatment; *J*_1_ is the initial steady flux of self-assembled membrane (L m^−2^ h^−1^); *J*_*n*_ is the recovered steady flux as *n* cycles (L m^−2^ h^−1^).

## 3. Results and discussion

### 3.1 Studies of UA composites

In this paper, UiO-66 was coated on the surface of γ-Al_2_O_3_ to obtain UA composites under optimal conditions (reaction time of 24 h, reaction temperature of 120 °C, *n*_ZrCl_4_ _:_ _*n*_H_2_O _:_ _*n*_H_2_BDC _:_ _*n*_DMF_ = 1 : 1_ _:_ _1 : 500). Then UA composites were further investigated and analyzed as described below.

#### 3.1.1 SEM and EDS studies of UA composites

**Fig. 5:**
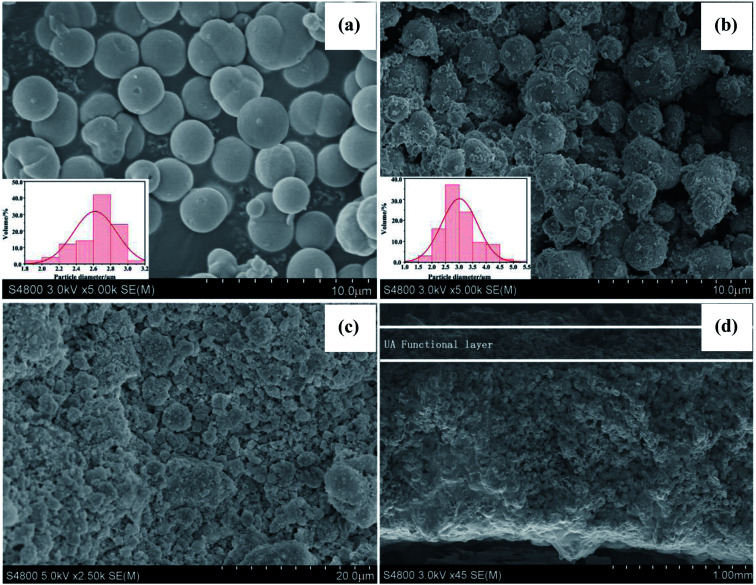
SEM image of Al_2_O_3_ particles (a). SEM image of UA composites (b). SEM images of surface (c) and cross-section (d) of UA self-assembled membrane. The formation condition of UA self-assembled membrane: membrane-forming pressure of 0.01 MPa, temperature of 25 °C.

Fig. 5(a) and (b) present the SEM images of γ-Al_2_O_3_ particles and UA composites, respectively. It can be observed that both γ-Al_2_O_3_ particles and UA composites have good sphericity and uniform size distributions. After modification, the mainly particle size distribution increases from 2.4–3.0 μm (γ-Al_2_O_3_ particles) to 2.5–3.5 μm (UA composites). And the surface of UA composites is not as smooth as γ-Al_2_O_3_ particles. This is because of the growth of UiO-66 on the surface of γ-Al_2_O_3_ particles. As shown in Fig. S2–S6 and Tables S2–S7,† the molar ratio of Al to Zr in UA particles is almost the same as that of Al to Zr in the synthetic process. Therefore, it could be inferred that UiO-66 has been successfully synthesized and coated on the surface of γ-Al_2_O_3_ particles.

#### 3.1.2 FTIR analysis of UA composites

**Fig. 6:**
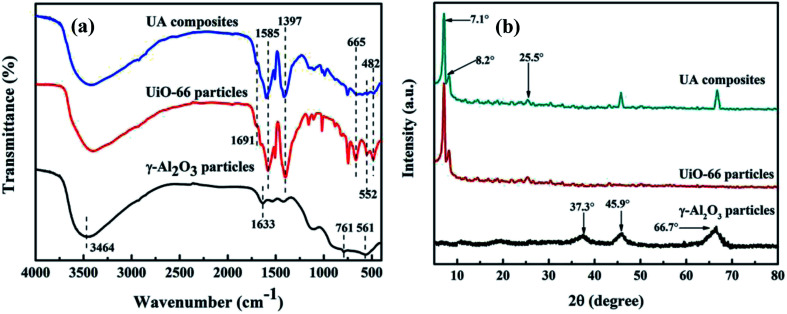
FTIR spectrum of functional materials (a). XRD patterns of functional materials (b). The UA composites were synthesized under optimal conditions: reaction time of 24 h, reaction temperature of 120 °C, *n*_ZrCl_4_ _:_ _*n*_H_2_O_ : *n*_H_2_BDC_ : *n*_DMF_ = 1 : 1 : 1 : 500.

Fig. 6(a) shows the FTIR spectra of γ-Al_2_O_3_ particles, UiO-66 particles and UA composites, respectively. The FTIR spectrum of γ-Al_2_O_3_ particles exhibit typical peaks at 761 and 561 cm^−1^, corresponding to the vibration model of AlO_6_.^32^ In addition, the peaks at around 3464 and 1633 cm^−1^ are ascribed to the stretching and bending vibrations of –OH groups. In the case of UiO-66 particles and UA composites, typical FTIR bands of UiO-66 are evident. The peak at 1691 cm^−1^ is ascribed to DMF, while the peaks at 1585 cm^−1^ and 1397 cm^−1^ are assigned to the in- and out-of-phase stretching modes of the carboxy group.^33^ It can be observed that in the spectrum of UA composites, the intensity of the carboxy group decreases to a large extent owing to the interaction between γ-Al_2_O_3_ and carboxy groups. Besides, the peaks at around 665, 552 and 482 cm^−1^ are attributed to the vibration of μ_3_-O stretch, Zr-(OC) asymmetric stretch and μ_3_-OH stretch vibration,^34,35^ further confirming the successful growth of UiO-66 on the surface of γ-Al_2_O_3_.

#### 3.1.3 XRD analysis of UA composites

XRD patterns of γ-Al_2_O_3_ particles, UiO-66 particles and UA composites are characterized and analyzed. As shown in Fig. 6(b), the diffraction peaks at 37.3°, 45.9° and 66.7° are the characteristic peaks of γ-Al_2_O_3_.^36^ The UiO-66 pattern has intense reflections at 2*θ* = 7.1°, 8.2°, and 25.5°, which is consistent with that reported for the UiO-66 topology.^33,37^ As for UA composites, the pattern exhibits similar diffraction peaks to those of UiO-66 particles, confirming the formation of UiO-66 crystals. Besides, UA composites have additional intense reflections at 2*θ* between 40–70°, which are typical of γ-Al_2_O_3_. Meanwhile, it can be also noted that γ-Al_2_O_3_ does not influence the crystallization of UiO-66 structures, but the intensity of the peaks in UA composites are lower than that of UiO-66, which is ascribed to strong interaction between γ-Al_2_O_3_ and carboxyl group.

### 3.2 SEM study of UA self-assembled membrane

Fig. 5(c) and (d) show the SEM micrographs of surface and cross section of UA self-assembled membrane under the optimal membrane-forming pressure (0.01 MPa). It can be observed from Fig. 5(c) and (d) that there is plentiful porous structure, rough and flat surface in the UA self-assembled membrane. The UA self-assembled membrane has asymmetric structure with a compact layer on the surface and a loose support layer beneath. As shown in Fig. 5(c) and (d), there are abundant channels existing in UA self-assembled membrane, which are more easier to form excellent filtration channels and enhance mass transfer process. Furthermore, an interface can be found between the support layer and functional layer. Above the interface is the functional layer, which may perform excellent separation and adsorption properties by size exclusion of membrane and adsorption effect UA composites. Below the interface is the diatomite support layer. The self-assembled membrane exhibits strong anti-compaction property and numerous tortuous filtration channels due to the high mechanical strength and good sphericity of diatomite particles. Therefore, the UA self-assembled membrane is desirable in cleaning oily seawater.

### 3.3 Optimum membrane-forming pressure of UA self-assembled membrane

**Fig. 7:**
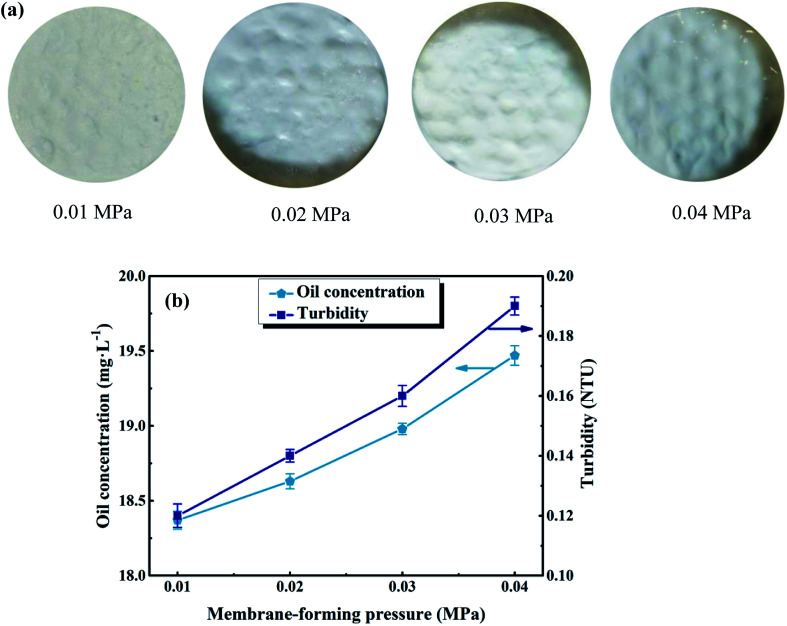
Effect of membrane-forming pressure on membrane surface morphology (a) and permeate water quality (b). Other parameters: treatment pressure is 0.1 MPa; membrane running time is 60 min; volume of oily seawater is 500 mL; temperature of feed solution is 25 °C.

In order to form a uniform UA self-assembled membrane for deep cleaning, the suitable membrane-forming pressure was investigated and confirmed. The experimental pressure was set at 0.01, 0.02, 0.03, and 0.04 MPa, respectively. As shown in Fig. 7(a), as the membrane-forming pressure increases, the uniformity of membrane becomes worse. This is because as the membrane-forming pressure increases, the flow of the fluid in the self-assembled membrane device becomes more unstable, making the surface of self-assembled membrane uneven. Besides, it can be seen from Fig. 7(b) that, oil concentration and turbidity of permeate increase as the forming pressure increasing. For instance, when the membrane-forming pressure is 0.04 MPa, the surface of membrane is uneven and the oil concentration, turbidity of permeate are 19.47 mg L^−1^ and 0.19 NTU, respectively. However, when the membrane-forming pressure reduces to 0.01 MPa, the surface of membrane is more uniform and the quality of permeate is much better with the oil concentration of 18.37 mg L^−1^ and turbidity of 0.12 NTU. We hypothesize this improvement is due to the fact that: (1) induced by the flow field under low membrane-forming pressure, numerous hydrogen bonding can be formed between the Zr–O sites, carboxyl of UiO-66 shell and the hydroxyl group of diatomite.^25^ (2) Uniform functional layer can be formed on the basis of diatomite support layer under the influence of hydrogen bonding. Therefore, the optimum membrane-forming pressure is 0.01 MPa.

### 3.4 The UA self-assembled membrane for oily seawater treatment

#### 3.4.1 Effect of different self-assembled membranes on permeate water quality

To evaluate the effect of different self-assembled membranes on membrane separation performance, the same mass of γ-Al_2_O_3_ and UA (the molar ratio of γ-Al_2_O_3_ to ZrCl_4_ is 4 : 1) were applied respectively to form functional layer on the diatomite support layer. Also, the diatomite support layer without functional layer was carried out for comparison. The experimental conditions were as follows: membrane-forming pressure of 0.01 MPa, treatment pressure of 0.1 MPa, temperature of 25 °C, membrane running time of 60 min and *V*_t_ of 500 mL.

The Table 2 compares the permeate quality and membrane resistance of diatomite self-assembled membrane with those of γ-Al_2_O_3_ and UA self-assembled membranes. To begin with, it can be observed that the membrane resistance of diatomite self-assembled membrane without functional layer is 0.34 × 10^11^ m^−1^. This is mainly due to its big particle size (50 μm). It is more easily for the oily seawater to pass through the membrane channels formed among diatomite. Besides, the γ-Al_2_O_3_ and UA self-assembled membranes exhibit much higher membrane resistance. The particle size distribution of these two types of function materials are mainly located at 3 μm. Thus, a compact functional layer can be formed, considerably resulting in much higher membrane resistance. However, the UA self-assembled membrane displays best performance with oil concentration of 18.37 mg L^−1^, SS concentration of 6.00 mg L^−1^ and turbidity of 0.12 NTU after membrane running for 60 min. As the introduction of UiO-66 into γ-Al_2_O_3_, the UA composites display better adsorption capacity, thus leading to lower oil and SS concentration in permeate.

**Table 2:** Effect of different self-assembled membranes on permeate water quality^a^

Self-assembled membrane	*C*_oil_ (mg L^−1^)	*C*_ss_ (mg L^−1^)	Turbidity (NTU)	*R* (×10^11^ m^−1^)
Diatomite	38.12	27.52	1.12	0.34
γ-Al_2_O_3_	29.97	8.61	0.40	9.46
UA	18.37	6.00	0.12	3.05

a *C*_oil_ is oil concentration; *C*_ss_ is suspended solid; *R* is membrane resistance, membrane running time is 60 min.

**Fig. 8:**
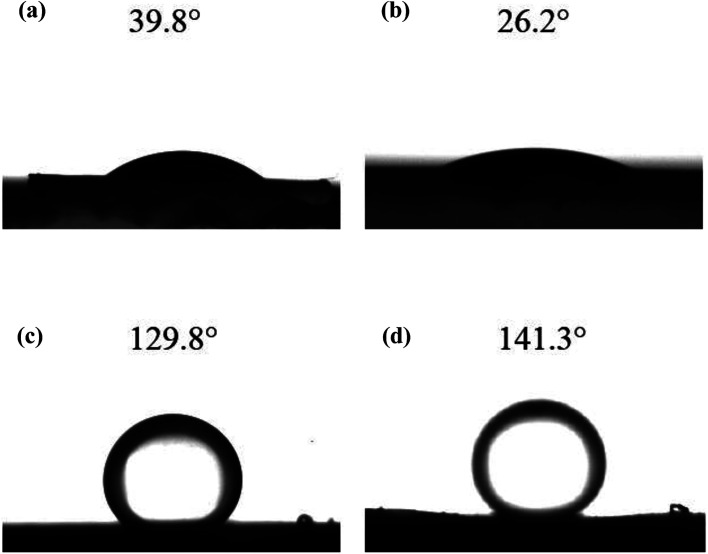
The water contact angles of γ-Al_2_O_3_ particles (a) and UA composites (b); and the under water oil contact angle of γ-Al_2_O_3_ functional layer (c) and UA functional layer (d).

As the hydrophilicity of functional materials has an important influence on membrane performance, water contact angle measurements were conducted to examine the hydrophilicity of functional materials. The water contact angles of γ-Al_2_O_3_ particles and UA composites are shown in Fig. 8(a) and (b), respectively. Compared with γ-Al_2_O_3_ particles (water contact angle is 39.8°) the hydrophilicity of UA composites is enhanced with water contact angle of 26.2°. As shown in Fig. 8(c) and (d), the underwater contact angles of γ-Al_2_O_3_ and UA functional layer are 129.8° and 141.3°, respectively. This results well show that the underwater oleophobicity of functional layer can be enhanced by improving the hydrophilic of functional materials. By enhancing the wetting properties of functional layer, the velocity of waste-water cycled and the anti-fouling property can be improved, which can reduce membrane running time and improve numbers of water cycled. Thus, the higher water yield and lower oil concentration in permeate can be obtained. Consistent with this, the instantaneous flux of UA self-assembled membrane is as high as 100.57 × 10^3^ L m^−2^ h^−1^, nearly 3.21 times that of γ-Al_2_O_3_ self-assembled membrane (31.34 × 10^3^ L m^−2^ h^−1^) at 30 min under same conditions. This demonstrates that the coatings of UiO-66 successfully improve the hydrophilicity of membrane.

#### 3.4.2 Effect of the molar ratio of γ-Al_2_O_3_ to ZrCl_4_ on permeate water quality

**Fig. 9:**
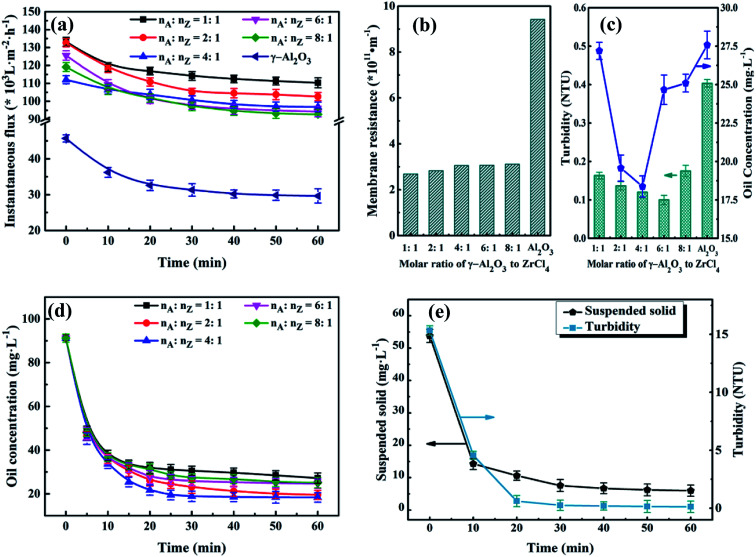
Effect of molar ratio of γ-Al_2_O_3_ to ZrCl_4_ on instantaneous flux (a), membrane resistance (b) and permeate quality (c); effect of membrane running time on permeate quality : oil concentration (d); suspended solid and turbidity (e), in which the molar ratio of γ-Al_2_O_3_ to ZrCl_4_ is 4 : 1.

The UA composites take the advantages of high APAM adsorption capacity of γ-Al_2_O_3_ and high hydrophilicity and excellent adsorption ability of UiO-66 crystals. The amount of UiO-66 coated on γ-Al_2_O_3_ surface is controlled by the precursor dosage and influences separation performance of UA self-assembled membrane. In order to determine the optimal molar ratio of γ-Al_2_O_3_ to UiO-66, different UA composites were synthesized by controlling molar ratio of γ-Al_2_O_3_ to ZrCl_4_ (*n*_A_ : *n*_Z_). The experiments were carried out under the condition described before. As shown in Fig. 9(a), the instantaneous fluxes of all membranes decline with membrane running time extends, and then reach stable when membrane running time reaches around 30 min. It can also be found that there is small difference among UA self-assembled membranes in terms of instantaneous flux, which is approximately 3.3 times as high as that of γ-Al_2_O_3_ self-assembled membrane (29.63 × 10^3^ L m^−2^ h^−1^) at 60 min. Correspondingly, as shown in Fig. 9(b), the resistance of UA self-assembled membranes is mainly distributed between 2.50 × 10^11^ and 3.50 × 10^11^ m^−1^, which is one third of that of γ-Al_2_O_3_ self-assembled membrane. This phenomenon can be attributed that the introduction of high hydrophilicity UiO-66 can improve instantaneous flux and reduce membrane resistance. Effect of UA microspheres with different UiO-66 coating amount on permeate water quality is shown in Fig. 9(c). Separation of the oily seawater is ascribed to size exclusion of membrane and adsorption effect of UA composites, including the synergistic effect of electric adsorption interactions between APAM and the uncovered positively charged γ-Al_2_O_3_, strong van der Waals force between *n*-alkanes and UiO-66, hydrogen-bonding interaction between iso-alkanes and the acid site of UiO-66, π–π interaction between aromatic hydrocarbons and the UiO-66 framework.^25,26^ The plausible mechanism of UA self-assembled membrane for treating oily seawater is shown in Fig. 3. Consequently, as *n*_A_ : *n*_Z_ increases, oil concentration and turbidity decrease first and then increase. When *n*_A_ : *n*_Z_ is 4 : 1, oil concentration is the lowest (18.37 mg L^−1^), and when *n*_A_ : *n*_Z_ increases to 6 : 1, turbidity reaches the minimum (0.10 NTU) after membrane running for 60 min. However, when *n*_A_ : *n*_Z_ is 6 : 1, the oil concentration is as high as 24.67 mg L^−1^. Therefore, take all the factors into consideration, the suitable molar ratio of γ-Al_2_O_3_ to ZrCl_4_ is 4 : 1.

#### 3.4.3 Effect of membrane running time on water quality of permeate

To improve the efficiency of water treatment, the optimum membrane running time was testified according to the permeate. Based on preceding results, the experimental conditions were determined: membrane-forming pressure of 0.01 MPa, treatment pressure of 0.1 MPa, temperature of 25 °C and *V*_t_ of 500 mL. Fig. 9(d) and (e) depict the variations of the water quality of the permeate with different membrane running time. As shown in Fig. 9(d), the oil concentration declines sharply in the initiate 30 min. Then it turns into a steady state in the next 30 min. As the membrane running time varies from 30 to 60 min, the water quality of the permeate just changed a bit. For instance, when the molar ratio of γ-Al_2_O_3_ to ZrCl_4_ is 4 : 1, oil concentration reaches to 18.90 mg L^−1^ at 30 min, 18.37 mg L^−1^ at 60 min. The observation demonstrates that the oil concentration does not decline considerably but keeps almost constant after another 30 min running. In other words, the latter 30 min in this experiment is unnecessary. Such similar phenomenon also appears in the Fig. 9(e). As for water yield shown in Table 3, its value reaches to 657.89 L m^−2^ h^−1^ after membrane running time for 30 min, which shows 131.57 and 95.99 L m^−2^ h^−1^ increase in contrast to PZSA^15^ and YSS^38^ self-assembled membrane, respectively. But, as the membrane running time increase from 30 to 60 min, the water yield decreases significantly from 657.89 to 328.95 L m^−2^ h^−1^. Hence, taking the water quality and water yield into account, the optimal membrane running time is set as 30 min.

**Table 3:** The water yield under different membrane running time^a^

The molar ratio of γ-Al_2_O_3_ to ZrCl_4_	*t*_t_ = 10 min		*t*_t_ = 30 min		*t*_t_ = 60 min	
	*C*_oil_ (mg L^−1^)	*Y*_w_ (L m^−2^ h^−1^)	*C*_oil_ (mg L^−1^)	*Y*_w_ (L m^−2^ h^−1^)	*C*_oil_ (mg L^−1^)	*Y*_w_ (L m^−2^ h^−1^)
1 : 1	37.59	1973.68	30.50	657.89	27.19	328.95
2 : 1	36.06	1973.68	23.09	657.89	19.57	328.95
4 : 1	33.62	1973.68	18.90	657.89	18.37	328.95
6 : 1	35.59	1973.68	25.82	657.89	24.67	328.95
8 : 1	36.78	1973.68	27.26	657.89	25.10	328.95

a *C*_oil_ is oil concentration; *Y*_w_ is water yield.

#### 3.4.4 Effect of treatment classes on permeate water quality

In order to satisfy the standard of discharge and recycle in China (SY/T5329-94, concentration of oil ≤ 8 mg L^−1^, SS ≤ 3 mg L^−1^) and explore the optimal cleaning effect, the same volume of oily seawater was applied under the optimum conditions determined before.

**Fig. 10:**
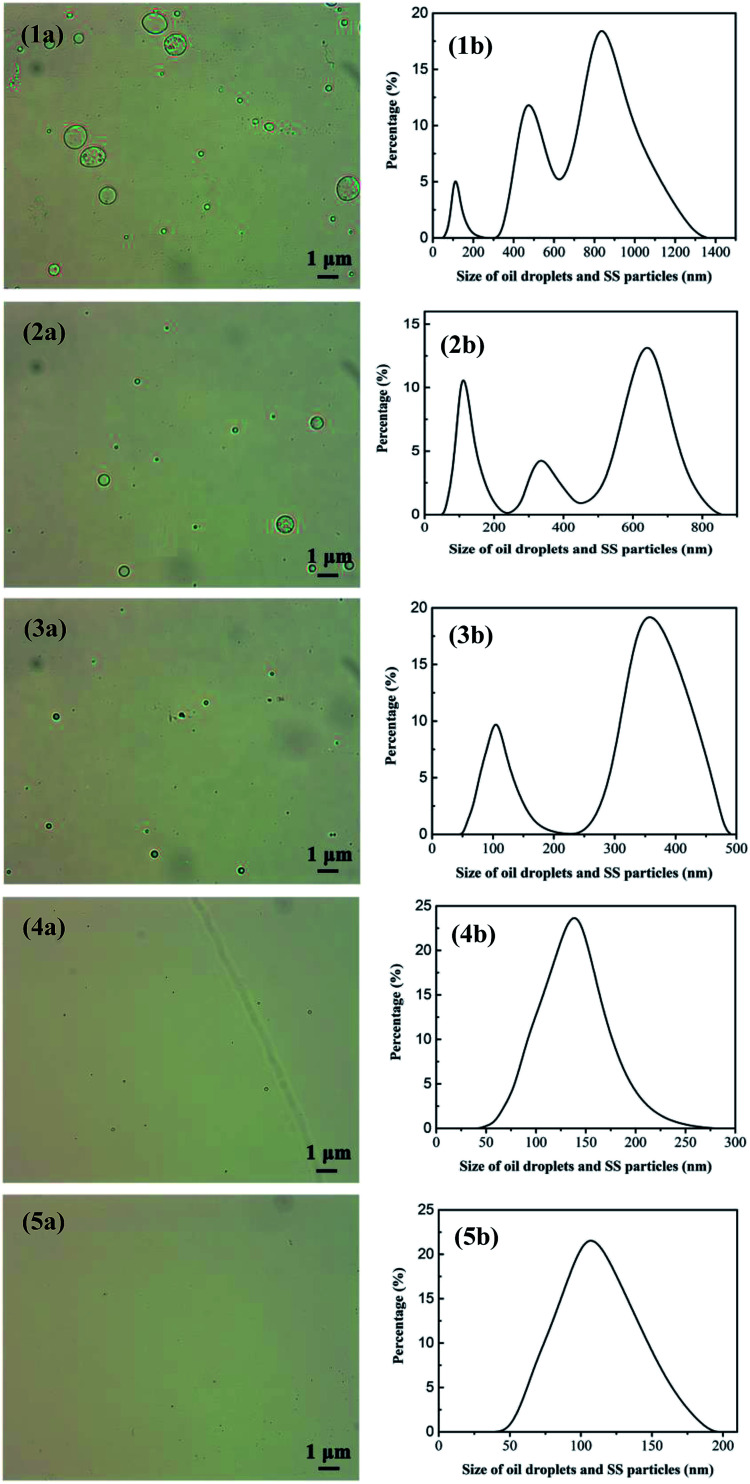
Size distribution of oil droplets and SS particles in the permeate after treatment classes reach 1, 2, 3, 4, 5; (1a), (2a), (3a), (4a) and (5a) are measured by optical microscope image. (1b), (2b), (3b), (4b), (5b) are tested *via* nano particle size analyzer.

Table 4 depicts the variation of permeate quality with different treatment classes. As shown in Table 4, obviously, as the treatment classes increase, the membrane running time decreases and the water yield significantly increases from 657.89 L m^−2^ h^−1^ (first class treatment) to 1233.55 L m^−2^ h^−1^ (fifth class treatment). What's more, the oil concentration dramatically declines from 91.22 to 18.90 mg L^−1^ in the first class treatment, and then gradually reduces to 2.61 mg L^−1^ as treatment classes reach 5, namely a final oil retention ratio of 97.14%. Compared with our previous work,^15,38^ oil retention ratio of UA self-assembled membrane is higher than that of PZSA self-assembled membrane of 96.58% and YSS self-assembled membrane of 95.51%. Furthermore, when treatment classes increase, SS decreases sharply from 53.75 to 2.69 mg L^−1^ in the 2 treatment classes, and then slowly reduces to 0.95 mg L^−1^ after 5 treatment classes. Fig. 10 shows the size distributions of oil droplets and SS particles in the permeate treated after different treatment classes. As shown in Fig. 10(1b), after 1 treatment class, the size of oil droplets and SS particles in permeate is mainly distributed at 50–250 nm and 300–1300 nm. By contrast, when the treatment classes reach 5, the size distribution of oil droplets and SS particles in permeate declines sharply and locates from 50 to 180 nm. As the treatment classes increase, the size distribution of membrane permeate decreases obviously, meanwhile, the retention ratio of oil droplets and SS particles increase. This phenomenon is due to that the pollutants in permeate can be constantly adsorbed and separated by the newly formed UA self-assembled membranes. When the treatment classes reach 2, the oil concentration and SS are 7.70 and 2.69 mg L^−1^, respectively, which satisfy the standard of discharge and recycle in China (SY/T5329-94, concentration of oil ≤ 8 mg L^−1^, SS ≤ 3 mg L^−1^) and the oily seawater can be discharged directly into the environment. Based on the above analysis, the UA self-assembled membranes have high flexibility in practical application, since the permeate water quality can be adjusted by changing classes to satisfy different requirement.

**Table 4:** Effect of treatment classes on membrane performance^a^

Treatment classes	*C*_oil_ (mg L^−1^)		*C*_ss_ (mg L^−1^)		*T*_t_ (min)	*Y*_w_ (L m^−2^ h^−1^)
	Before running	After running	Before running	After running		
1	91.22	18.90	53.75	7.58	30	657.89
2	18.90	7.70	7.58	2.69	24	822.37
3	7.70	5.00	2.69	1.88	20	986.84
4	5.00	3.80	1.88	1.38	17	1160.99
5	3.80	2.61	1.38	0.95	16	1233.55

a *C*_oil_ is oil concentration; *C*_ss_ is suspended solid; *T*_t_ is membrane running time; *Y*_w_ is water yield.

#### 3.4.5 The recycle of self-assembled membrane

**Fig. 11:**
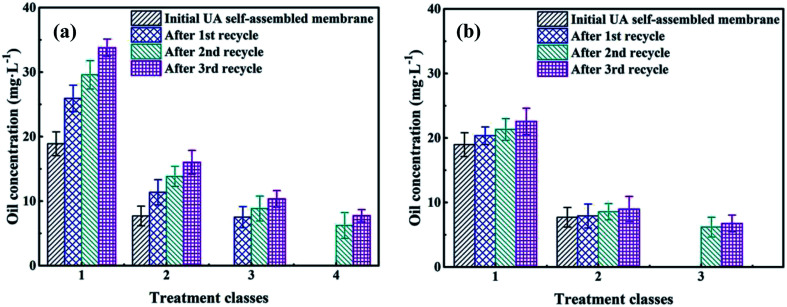
The recycle performance of self-assembled membrane: without hydraulic cleaning (a); with hydraulic cleaning (b).

To save resources, reduce cost and decline secondary pollution, the membrane materials need to be recycled. The mixture of UA self-assembled membrane materials (diatomite and UA composites) and the trapped pollutants were calcined under 200 °C. Because most of the pollutants can be decomposed and removed at 200 °C, UiO-66 still maintains its inherent crystal and structure at such temperature. As shown in Fig. 11(a), oil concentration of the permeate water declines as treatment class extends for the self-assembled membranes that were directly formed by recycled materials. However, the separation performance of the recycled membranes is lower than that of the fresh UA self-assembled membrane, especially as recycle number increases. This is because some fragment of long-chain alkane and SS still remain on the surface of recycled materials, and the hardening of these residual substance limits the full contact between functional particles and oily seawater. Therefore, the membrane materials are washed using water before forming UA self-assembled membrane. The treatment of oily seawater with UA self-assembled membrane formed by recycled membrane materials with hydraulic cleaning are shown in Fig. 11(b). It can be seen that hydraulic cleaning can effectively improve membrane performance. After treated by UA self-assembled membrane formed by recycled membrane materials (1st recycle) with hydraulic cleaning, the seawater can satisfy discharge and recycle standard in China as the treatment class extends to 2. However, it needs 3 treatment classes to satisfy the standard if the UA self-membranes are directly formed by recycled materials (1st recycle). Meanwhile, when the recycling number of membrane materials increases to 2 and 3, hydraulic cleaning still shows significant influence on membrane performance, which shortens the treatment classes to satisfy the discharge and recycle standard.

#### 3.4.6 Long-term experiment of self-assembled membrane

**Fig. 12:**
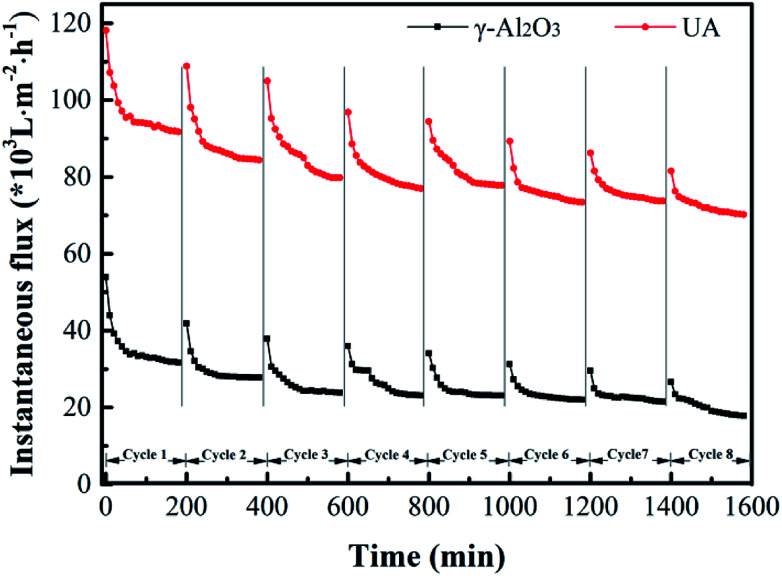
Variation of instantaneous flux of the γ-Al_2_O_3_ and UA self-assembled membranes for 8 cycles oily seawater treatment.

To study the membrane fouling over long-term running, the γ-Al_2_O_3_ and UA self-assembled membranes were implemented to filtrate oily seawater for continuous 8 cycles. Each cycle lasted 3 hours. At the interval of each cycle, the fouled membrane materials were calcined under 200 °C for 2 hours. As shown in Fig. 12 and Table 5, with the increase of cycles, the flux recovery ratio decreases gradually. For UA self-assembled membrane, the flux recovery ratio is approximately 91.4% as 2 cycles ended and 87.63% as 3 cycles ended. It is a normal phenomenon because of the irreversible fouling. Besides, the flux recovery ratio decline of UA self-assembled membrane is less evident than that of γ-Al_2_O_3_ self-assembled membrane. After 8 cycles treatment, the flux recovery ratio of UA self-assembled membrane is 76.34%, whereas the flux recovery ratio of γ-Al_2_O_3_ self-assembled membrane is 60.24%. The change in oil retention ratio is consistent with the trend of flux recovery ratio. This phenomenon well indicated that the introduction of high hydrophilic UiO-66 can significantly elevate the anti-fouling property of self-assembled membrane.

**Table 5:** Flux recovery ratio (FRR) and oil retention ratio (ORR) of each cycle in long-term experiment

	Self-assembled membrane	1st	2nd	3rd	4th	5th	6th	7th	8th
FRR (%)	γ-Al_2_O_3_	—	84.33	73.80	70.78	69.58	67.77	66.27	60.24
	UA	—	91.4	87.63	84.41	83.87	81.18	79.89	76.34
ORR (%)	γ-Al_2_O_3_	78.49	77.02	74.94	72.32	68.73	64.36	60.91	58.80
	UA	88.69	85.79	84.08	82.17	79.65	76.75	73.31	69.33

## 4. Conclusion

In this work, the UA self-assembled membrane formed under optimum conditions (membrane-forming pressure of 0.01 MPa, treatment pressure of 0.10 MPa, temperature of 25 °C and *V*_t_ of 500 mL) performs attractive oily seawater cleaning property. After 2 treatment classes, the concentration of oil and suspended solid are 7.70 and 2.69 mg L^−1^, respectively, which satisfy the standard of discharge and recycle in China. Furthermore, UA self-assembled membrane can be recycled by calcination, and the membrane performance can be improved by hydraulic cleaning. Lastly, the UA self-assembled membrane shows decent stability and durability in long-term running. The results indicate that UA self-assembled membrane performs high hydrophilicity, low membrane resistance, desirable separation, selective adsorption properties and decent durability in oily seawater treatment. Therefore, UA self-assembled membrane has attractive application prospects in effectively cleaning oily seawater.

## Conflicts of interest

There are no conflicts to declare.

## Supplementary Material

RA-009-C9RA00521H-s001
