# Expression of concern: Self-assembled membrane manufactured by metal–organic framework (UiO-66) coated γ-Al_2_O_3_ for cleaning oily seawater

**DOI:** 10.1039/d2ra90017c

**Published:** 2022-02-17

**Authors:** Laura Fisher

**Affiliations:** Royal Society of Chemistry Thomas Graham House, Science Park, Milton Road Cambridge UK

## Abstract

Expression of concern for ‘Self-assembled membrane manufactured by metal–organic framework (UiO-66) coated γ-Al_2_O_3_ for cleaning oily seawater’ by Cunlong Li *et al.*, *RSC Adv*., 2019, **9**, 10702–10714, DOI: 10.1039/C9RA00521H.

The following article ‘Self-assembled membrane manufactured by metal–organic framework (UiO-66) coated γ-Al_2_O_3_ for cleaning oily seawater’ has been published in *RSC Advances*.

The XRD data shown in Fig. 6b is the same for the UA composites (shown in green) and UiO-66 particles (shown in red), with the exception of two peaks that are not present in the UiO-66 pattern.

In Fig. 7a, the panel for 0.03 MPa is a rotated and processed duplicate of the panel for 0.02 MPa.

The authors were contacted for comment and asked to provide raw data but have not responded to these concerns. *RSC Advances* is publishing this Expression of Concern to alert readers to the concerns raised. An Expression of Concern will continue to be associated with the article until we receive conclusive evidence regarding the reliability of the reported data.

Laura Fisher

11^th^ February 2022

Executive Editor, *RSC Advances*

## Supplementary Material

