# Update to expression of concern: Self-assembled membrane manufactured by metal–organic framework (UiO-66) coated γ-Al_2_O_3_ for cleaning oily seawater

**DOI:** 10.1039/d2ra90115c

**Published:** 2022-11-15

**Authors:** Cunlong Li, Yuqing Zhang, Ming Yong, Wei Liu, Jiaqi Wang

**Affiliations:** School of Chemical Engineering and Technology, Tianjin University Tianjin 300072 PR China zhangyuqing@tju.edu.cn +86-22-27403389 +86-13-602077041

## Abstract

Update to expression of concern for ‘Self-assembled membrane manufactured by metal–organic framework (UiO-66) coated γ-Al_2_O_3_ for cleaning oily seawater’ by Cunlong Li *et al.*, *RSC Adv.*, 2019, **9**, 10702–10714, https://doi.org/10.1039/C9RA00521H.

The following article ‘Self-assembled membrane manufactured by metal–organic framework (UiO-66) coated γ-Al_2_O_3_ for cleaning oily seawater’ has been published in *RSC Advances*.

*RSC Advances* published an expression of concern (https://doi.org/10.1039/D2RA90017C) in order to alert our readers to the fact that the Royal Society of Chemistry had been provided with credible information suggesting that the results presented in this paper, in particular Fig. 6 and 7, may not be reliable.

We are now able to provide the following update:

The authors do not have the original raw data for Fig. 6b so they have repeated their experiments to characterise UiO-66, γ-Al_2_O_3_ and UiO-66 coated γ-Al_2_O_3_ particles by XRD.

The authors have stated that in Fig. 7a the panel for 0.02 MPa was accidentally used to represent 0.03 MPa. The authors have provided the original correct data for 0.03 MPa.

The Royal Society of Chemistry has asked the affiliated institution (Tianjin University) to investigate this matter and confirm the integrity and reliability of the new data and figures provided.

An expression of concern will continue to be associated with this manuscript until we receive information from the institution on this matter.

Laura Fisher

10th November 2022

Executive Editor, *RSC Advances*

## Supplementary Material

