# Supplementary material for: Self-assembled membrane manufactured by metal–organic framework (UiO-66) coated γ-Al2O3 for cleaning oily seawater
Source: RSC Adv. 2019 Apr 5;9(19):10702–14. doi: 10.1039/c9ra00521h (PMC9062490; doi:10.1039/c9ra00521h)
Supplement: RA-009-C9RA00521H-s001 [file RA-009-C9RA00521H-s001.pdf]

Electronic Supplementary Material (ESI) for RSC Advances.
This journal is © The Royal Society of Chemistry 2019

Supporting Information

Self-assembled membrane manufactured by metal-organic framework (UiO-66) coated γ-Al_2O_3 for cleaning oily seawater

Cunlong Li[1], Yuqing Zhang[1], Ming Yong[1], Wei Liu[1], Jiaqi Wang[1]*

[1]School of Chemical Engineering and Technology, Tianjin University, Tianjin 300072, PR China

Corresponding Author

*E-mail: zhangyuqing@tju.edu.cn

Synthesis of UiO-66:

0.5 mmol $ZrCl_4$, 0.5 mmol H_2BDC and 0.5 mmol deionized water were dissolved in 250 mmol DMF under stirring. The mixture solution was then transferred to Teflon-lined stainless steel autoclaves. The reaction was then carried out at 120 ℃ for 24 h. After being cooled to room temperature, the product was washed by DMF and methanol repeatedly. The powder was then dried under oven condition at 120 ℃ overnight for further use.

Hydraulic cleaning process

The recycled membrane materials were added to the coating solution tank with 500 ml reused water under stirring. The coating solution prepared by recycled membrane materials was pumped into the self-assembled membrane module with porous support. The membrane was gradually formed on the porous layer as coating solution cycles in the self-assembled membrane system. After the recycled membrane was formed, the reused water cycles for another 15 min in the self-assembled membrane system. Subsequently, we disassembled the self-assembled membrane module and took out the self-assembled membrane. The self-assembled membrane formed by recycled materials was dried in vacuum oven until constant weight at 80 ℃ for further use.

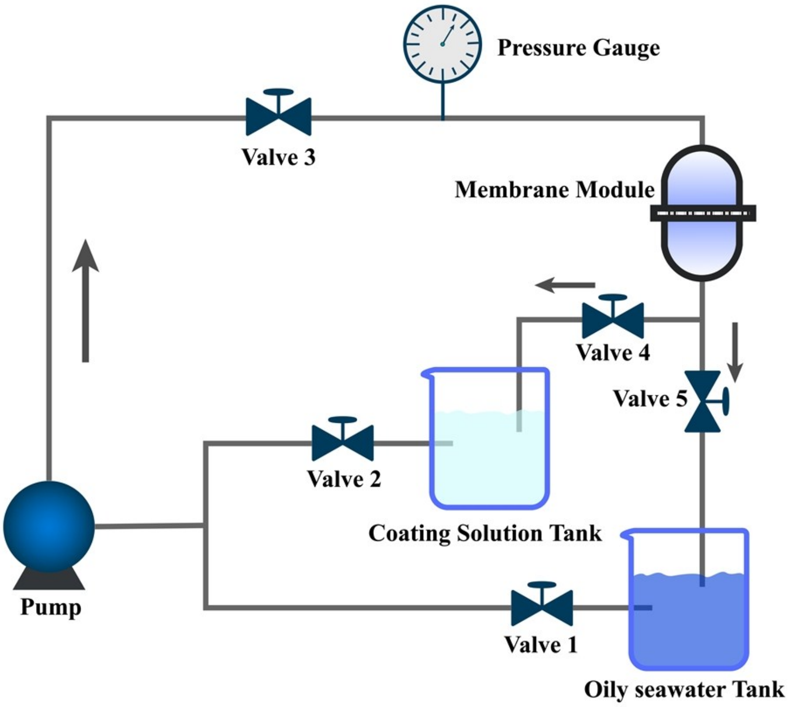

Fig. S1. Schematic diagram of experiment setup.

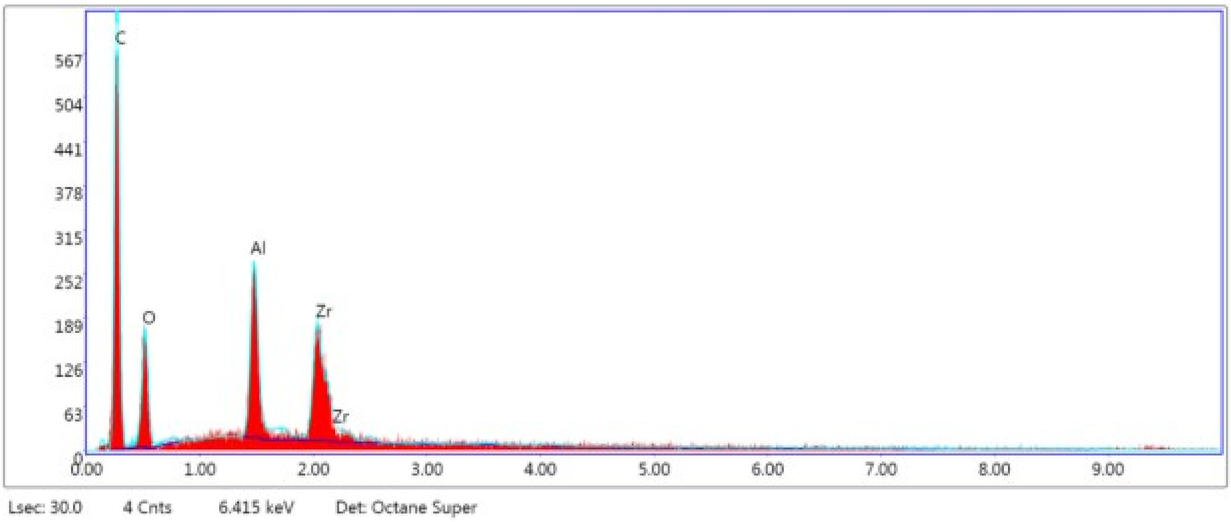

Fig. S2. EDS spectrum of UA composites (n_A: n_Z= 1: 1, "n_A: n_Z" represents the molar ratio of γ-Al_2O_3 to $ZrCl_4$ in the process of synthesizing UA composites.)

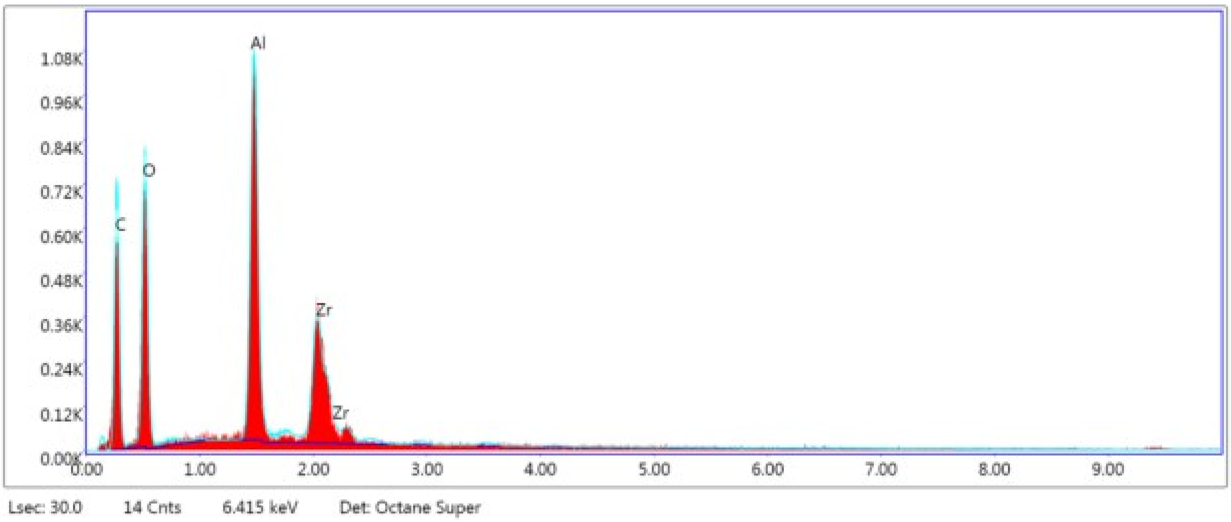

Fig. S3. EDS spectrum of UA composites (n_A: n_Z= 1: 1, "n_A: n_Z" represents the molar ratio of γ-Al_2O_3 to $ZrCl_4$ in the process of synthesizing UA composites.)

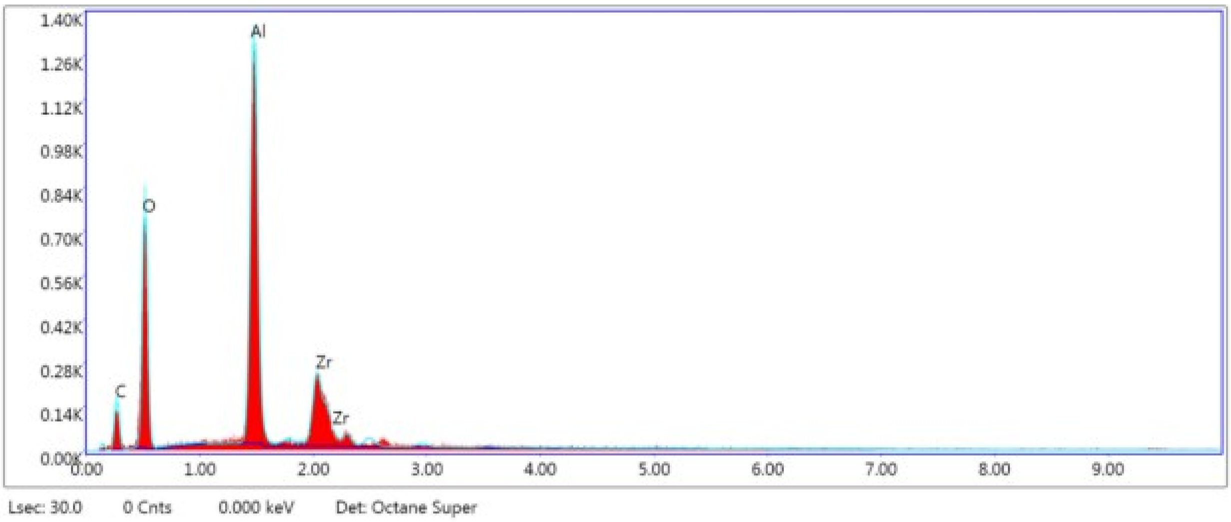

Fig. S4. EDS spectrum of UA composites (n_A: n_Z= 1: 1, “n_A: n_Z” represents the molar ratio of γ-Al_2O_3 to $ZrCl_4$ in the process of synthesizing UA composites.)

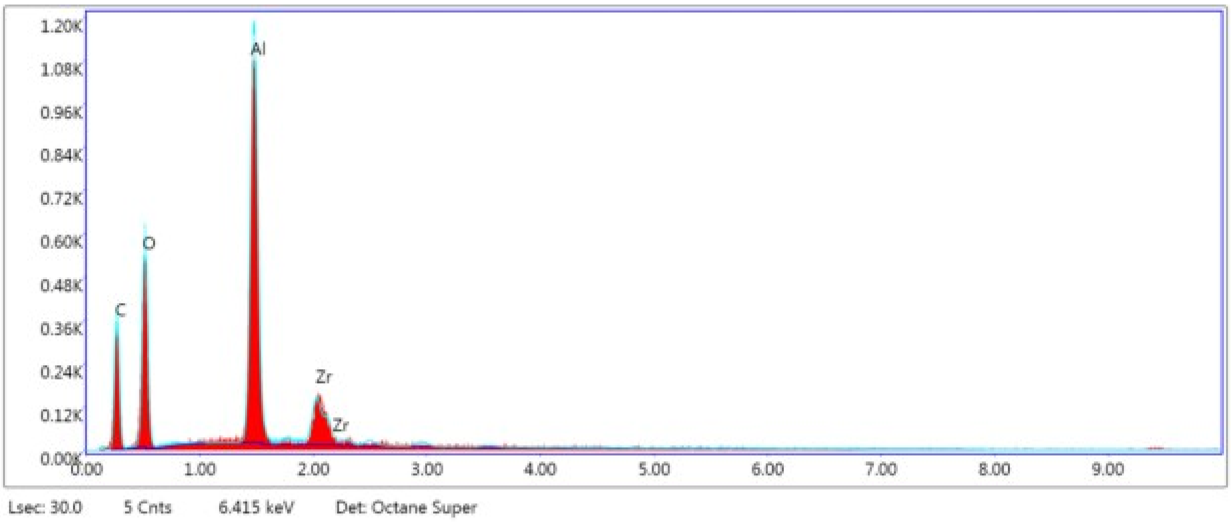

Fig. S5. EDS spectrum of UA composites (n_A: n_Z= 1: 1, "n_A: n_Z" represents the molar ratio of γ-Al_2O_3 to $ZrCl_4$ in the process of synthesizing UA composites.)

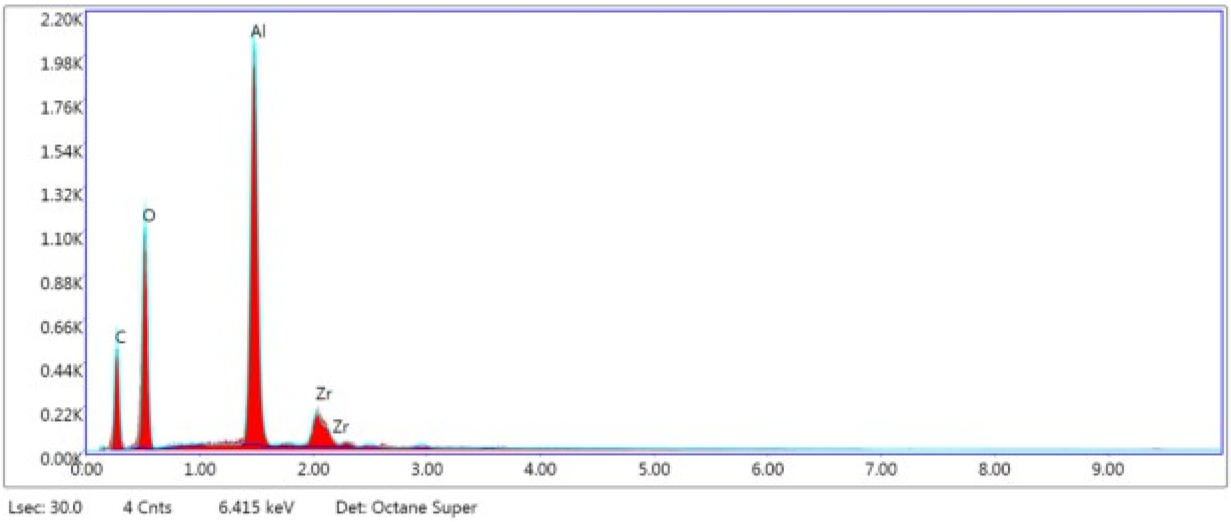

Fig. S6. EDS spectrum of UA composites (n_A: n_Z= 1: 1, "n_A: n_Z" represents the molar ratio of γ-Al_2O_3 to $ZrCl_4$ in the process of synthesizing UA composites.)

Table S1.

The detailed composition of diatomite.

Component (Units)	Concentration
SiO_2 (wt%)	≧85.0
Al_2O_3 (wt%)	≦5.0
Fe_2O_3 (wt%)	≦2.0
CaO (wt%)	≦1.0
MgO (wt%)	≦0.9
pb (mg·L-1)	8

Table S2.

The relative content of elements in UA composites (n_A: n_Z= 1: 1)

Element	Weight %	Atomic %
C K	62.60	77.09
O K	18.42	17.03
Al K	7.26	3.98
Zr L	11.72	1.90
Total	100.00	100.00

* "n_A: n_Z" represents the molar ratio of γ-Al_2O_3 to $ZrCl_4$ in the process of synthesizing UA composites.

Table S3.

The relative content of elements in UA composites (n_A: n_Z= 2: 1)

Element	Weight %	Atomic %
C K	39.73	54.30
O K	33.90	34.78
Al K	14.41	8.77
Zr L	11.96	2.15
Total	100.00	100.00

* "n_A: n_Z" represents the molar ratio of γ-Al_2O_3 to $ZrCl_4$ in the process of synthesizing UA composites.

Table S4.

The relative content of elements in UA composites (n_A: n_Z= 4: 1)

Element	Weight %	Atomic %
C K	19.54	30.42
O K	41.03	47.95
Al K	27.76	19.24
Zr L	11.67	2.39
Total	100.00	100.00

* "n_A: n_Z" represents the molar ratio of γ-Al_2O_3 to $ZrCl_4$ in the process of synthesizing UA composites.

Table S5.

The relative content of elements in UA composites (n_A: n_Z= 6: 1)

Element	Weight %	Atomic %
C K	35.99	49.26
O K	34.66	35.61
Al K	22.94	13.98
Zr L	6.41	1.15
Total	100.00	100.00

* "n_A: n_Z" represents the molar ratio of γ-Al_2O_3 to $ZrCl_4$ in the process of synthesizing UA composites.

Table S6.

The relative content of elements in UA composites (n_A: n_Z= 8: 1)

Element	Weight %	Atomic %
C K	32.57	44.84
O K	38.15	39.43
Al K	24.16	14.80
Zr L	5.12	0.93
Total	100.00	100.00

* "n_A: n_Z" represents the molar ratio of γ-Al_2O_3 to $ZrCl_4$ in the process of synthesizing UA composites.

Table S7.

The molar ratio of Al to Zr (N_{Al}: N_{Zr}) in UA composites with different n_A: n_Z

Experimental condition		EDS analysis
n_A: n_Z	N_{Al}: N_{Zr}	N_{Al}: N_{Zr}
1: 1	2: 1	2.09: 1
2: 1	4: 1	4.08: 1
4: 1	8: 1	8.05: 1
6: 1	12: 1	12.16: 1
8: 1	16: 1	15.91: 1

* "n_A: n_Z" represents the molar ratio of γ-Al_2O_3 to $ZrCl_4$ in the process of synthesizing UA composites.
